# Modelling the vascular response to sympathetic postganglionic nerve activity

**DOI:** 10.1016/j.jtbi.2015.01.037

**Published:** 2015-04-21

**Authors:** Linford J.B. Briant, Julian F.R. Paton, Anthony E. Pickering, Alan R. Champneys

**Affiliations:** a School of Physiology & Pharmacology, Medical Sciences Building, University Walk, University of Bristol, Bristol BS8 1TD, UK; b Department of Engineering Mathematics, Merchant Venturers Building, Woodland Road, University of Bristol, Bristol BS8 1UB, UK; c Department of Anaesthesia, University Hospitals Bristol, Bristol BS2 8HW, UK

**Keywords:** Calcium dynamics, Bursting, Hypertension, Sympathetic nervous system, Neurone dynamics

## Abstract

This paper explores the influence of burst properties of the sympathetic nervous system on arterial contractility. Specifically, a mathematical model is constructed of the pathway from action potential generation in a sympathetic postganglionic neurone to contraction of an arterial smooth muscle cell. The differential equation model is a synthesis of models of the individual physiological processes, and is shown to be consistent with physiological data.

The model is found to be unresponsive to tonic (regular) stimulation at typical frequencies recorded in sympathetic efferents. However, when stimulated at the same average frequency, but with repetitive respiratory-modulated burst patterns, it produces marked contractions. Moreover, the contractile force produced is found to be highly dependent on the number of spikes in each burst. In particular, when the model is driven by preganglionic spike trains recorded from wild-type and spontaneously hypertensive rats (which have increased spiking during each burst) the contractile force was found to be 10-fold greater in the hypertensive case. An explanation is provided in terms of the summative increased release of noradrenaline. Furthermore, the results suggest the marked effect that hypertensive spike trains had on smooth muscle cell tone can provide a significant contribution to the pathology of hypertension.

## Highlights

• We model the sympathetic-driven contraction of a vascular smooth muscle cell.
• The cell is unresponsive to tonic stimulation at typical sympathetic frequencies.
• We quantify the force produced by the cell in response to sympathetic bursting.
• The response of the cell is strongly dependent on burst amplitude and duration.
• Recordings from hypertensive animals produce significant contractile forces.

## 1 Introduction

The contractile behaviour of smooth muscle cells (SMCs) in resistance arteries is known to be controlled by the sympathetic nervous system, whose activity exhibits bursting rhythms. In particular, the activity of sympathetic postganglionic neurones innervating such cells comprises intermittent bursts of varying amplitude and frequency (Malpas, 1998). The bursting discharge drives arterial smooth muscle cell contractions, causing vasoconstriction and increases in arterial blood pressure. This activity, in both pre- and postganglionic neurones, is known to exhibit bursting that is entrained to the respiratory rhythm, due to central coupling with respiratory pattern generators (Habler et al., 1994). A quantification of how this bursting activity influences arterial smooth muscle cell contractility is important as changes to sympathetic bursting are characteristic of cardiovascular diseases, such as hypertension and heart failure. This sympathetic nerve activity is known to be elevated in the spontaneously hypertensive (SH) rat and to exhibit amplified sympathetic-respiratory coupling (Simms et al., 2009). Recently, intracellular studies in the SH rat have shown that preganglionic neurones exhibit amplified respiratory modulation in their discharge pattern (vs normotensive controls; Wistar–Kyoto rats, WKY), characterised by 2–3 more action potentials per respiratory burst (Briant et al., 2014). However, this amounts to a relatively small (≈1 Hz) increase in average firing frequency, which is still below the threshold for a mechanical response in many sympathetic effectors (Nilsson et al., 1985; Lacroix et al., 1988; Pernow et al., 1989). Because of this, the contribution of the amplified respiratory-sympathetic coupling to vasoconstriction and the pathology of hypertension is unclear. As we shall see in this paper though, it is not the average firing frequency alone that accounts for change in SMC contractility.

Specifically, to investigate and quantify the influence of sympathetic bursts on the degree of smooth muscle cell tone, we have built a mathematical model of sympathetic transmission to a single arterial smooth muscle cell (SMC). Several sets of differential equations are coupled to provide a pathway from action potential generation in a sympathetic postganglionic neurone (Briant et al., 2014) to noradrenaline release from a postganglionic terminal (Yamada and Zucker, 1992), intra-SMC signalling (Li and Rinzel, 1994; Fink et al., 1999; Lemon et al., 2003; Bennett et al., 2005) and the subsequent cross-bridge formation and contractile force produced by the SMC (Hai and Murphy, 1988). In particular, the latter stages of the model adopts the differential equation model described by Bennett and co-workers (Lemon et al., 2003; Bennett et al., 2005) for the behaviour of SMCs surrounding the rat tail artery. Our model begins with the neural signalling and the consequential release of noradrenaline, that drives this SMC model. We have also revised the assumptions and physiological parameters from Bennett et al. (2005) in the light of physiological data (Julien et al., 2001). This has enabled us to investigate the influence of the pattern of sympathetic postganglionic neurone activity on the contraction of arterial SMCs.

The rest of this paper is outlined as follows. Section 2 provides an overview of the model we have developed, highlighting the key assumptions made and giving reference to the appropriate literature where the individual components of the model were developed. Section 3 presents the simulation methodology and any adaptations to the models. Section 4 contains the simulation results, both those that were used to check the modelling parameters and assumptions against previously published data and also the simulation of the entire pathway. Care is taken to quantify the contractile force observed as a function of various input burst parameters. Finally, Section 5 discusses the results of our modelling in context of the literature and proposes novel hypotheses to be tested experimentally.

## 2 Model overview

**Fig. 1:**
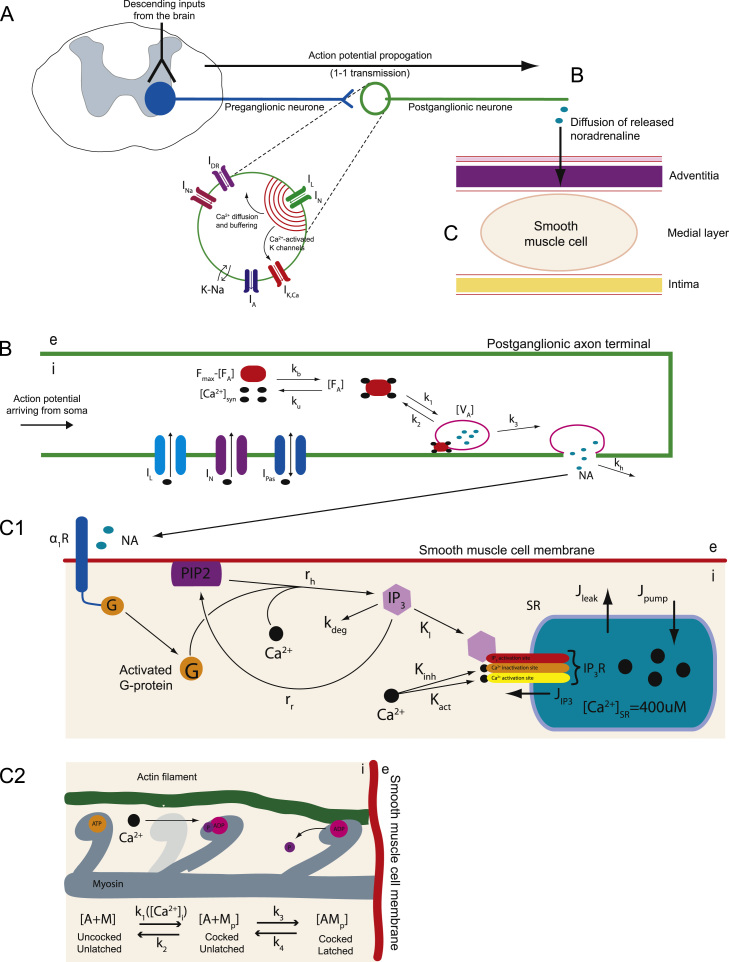
The mathematically modelled pathway from SPGN excitation to smooth muscle cell activation. The mathematically modelled pathway from SPGN excitation to smooth muscle cell activation. (A) The model of a SPGN from Briant et al. (2014). Action potentials propagate down the SPGN axon to the postganglionic terminal. (B) This activates *I*_*L*_ and *I*_*N*_, triggering the influx of calcium into the postganglionic terminal, increasing ${\left[{\mathrm{Ca}}^{2+}\right]}_{\mathrm{syn}}$. Four molecules of intracellular calcium bind to a fusion protein, activating it (*F*_*A*_). Once activated, the fusion protein can bind to, and consequently activate, a vesicle *V*. The activated vesicles, *V*_*A*_, are assumed to be pre-docked to the synaptic membrane; once activated, it immediately releases its NA contents into the cleft. (C_1_) Released noradrenaline activates *α*_1_Rs on the SMC membrane, activating a G-protein (G). This G-protein, drives the hydrolysis of PIP_2_. Hydrolysed PIP2 cleaves to form IP3, which activates an IP_3_R located on the membrane of the SR. Activation of this receptor causes an efflux of Ca^2+^ from the SR (*J*_*IP*3_), increasing ${\left[{\mathrm{Ca}}^{2+}\right]}_{\mathrm{SMC}}$. These receptors also have inactivation and activation sites for ${\left[{\mathrm{Ca}}^{2+}\right]}_{\mathrm{SMC}}$. Fluxes of Ca^2+^ across the SR membrane also exist due to leakage (*J*_*leak*_) and calcium pumps (*J*_*pump*_). (C_2_) The intra-SMC matrix contains actin (A) and myosin (M) filaments. At rest these filaments are in a detached state, A+M. When the myosin heads are phosphorylated by calcium (*M*_*P*_), they able to attach to the actin filaments, yielding the state *AM*_*P*_—a cross-bridge. This cross-bridge can then conduct a ‘power stroke’, sliding the actin filament and producing a contractile force.

Fig. 1 summarises the neurovascular pathway modelled—the transmission of information from a sympathetic postganglionic neurone (SPGN) to an arterial SMC. The model considers separately the mechanisms of action potential generation in a SPGN (Fig. 1A), its transmission to the distal end of the axon, causing release of noradrenaline (NA) from the axon terminal into the neuromuscular junction (Fig. 1B), the subsequent activation of the SMC triggering contraction (Fig. 1C).

This model is novel in that it investigates the of influence sympathetic output on SMC contraction at a cellular level, and can relate sympathetic discharge patterns to contractile response. The system includes a model of a sympathetic neurone (Briant et al., 2014) coupled to a model of neurotransmitter release (Destexhe et al., 1994). The released noradrenaline is used to drive a model of SMC contraction (Bennett et al., 2005). By modelling the vasoconstrictor processes at this level of detail, the influence of sympathetic patterning on arterial smooth muscle cell contractility can for the first time be fully quantified and add to our understanding of increased vascular tone in pathological conditions such as hypertension.

### 2.1 Postganglionic action potential generation and propagation

Action potentials are generated in the sympathetic postganglionic neurone. We use a Hodgkin–Huxley style partial differential equation model of a sympathetic neurone that was reported by Briant et al. (2014) and implemented in NEURON (Carnevale and Hines, 2006). The code for this model has been deposited on ModelDB (senselab.med.yale.edu/modeldb; accession number: 151482). The model is represented schematically in Fig. 1A, and described in brief as follows. A single un-branched axon emerges from the soma (length 500μm and diameter of 0.5μm, 20 segments), ending at the site of release of NA. The ion channel currents included in the model are depicted graphically in Fig. 1A. These currents are all present in the soma, with the exception of the leak (*I*_*pas*_) that is present throughout the cell membrane. The axon has the Hodgkin–Huxley conductances *I*_*Na*_, *I*_*DR*_ required for spike generation and also includes N- and L-type calcium currents *I*_*N*_ and *I*_*L*_, because these channels are known to drive synaptic transmission at sympathetic nerve-endings (Rittenhouse and Zigmond, 1999). Spiking in the neurone model was driven by the injection of current pulses (2nA×2ms) into the soma.

The calcium current at the distal end of the axon, generated in response to action potentials arriving from the soma of the neurone model, is used to drive release of NA at the neuromuscular junction. An action potential arriving at the axon terminal of the postganglionic model activates high-threshold voltage-sensitive calcium currents *I*_*N*_ and *I*_*L*_. This calcium influx locally elevates the intracellular concentration of calcium at the terminal/synapse of the axon, ${\left[{\mathrm{Ca}}^{2+}\right]}_{\mathrm{syn}}$. Here and in what follows, square brackets [·] are used to describe the concentration of a chemical species, with a subscript, in this case _*syn*_, indicating a particular sub-concentration.

The kinetics of this sympathetic calcium is determined by the calcium fluxes across the membrane of the synapse ($I_{\mathrm{Ca}}=I_{N}+I_{L}$ and the calcium pumps) and by calcium buffering:

(1) $$\frac{d{\left[{\mathrm{Ca}}^{2+}\right]}_{\mathrm{syn}}}{\mathrm{dt}}=-\frac{I_{\mathrm{Ca}}}{2F_{d}d}-\frac{k_{t}{\left[{\mathrm{Ca}}^{2+}\right]}_{\mathrm{syn}}}{{\left[{\mathrm{Ca}}^{2+}\right]}_{\mathrm{syn}}+K}+\frac{{\left[{\mathrm{Ca}}^{2+}\right]}_{\infty }-{\left[{\mathrm{Ca}}^{2+}\right]}_{\mathrm{syn}}}{\tau_{r}}.$$

Here, *F*_*d*_ is Faraday׳s constant and *d* is the depth of the hemispherical calcium domain. *I*_*Ca*_ is the sum of all calcium currents across the membrane $I_{N}+I_{L}$, extracted from the distal end of the axon in the SPGN model. The pumping of calcium across the membrane occurs at a rate *k*_*t*_ with Michaelis constant *K*. Calcium is buffered with time-constant *τ*_*r*_ to a concentration ${\left[{\mathrm{Ca}}^{2+}\right]}_{\infty }$. The calcium concentration ${\left[{\mathrm{Ca}}^{2+}\right]}_{\mathrm{syn}}$ drives the release of NA.

### 2.2 Noradrenaline release kinetics

Yamada and Zucker (1992) constructed a differential equation model of transmitter release from a neurone terminal, based on a model by Parnas and Parnas (1988). These kinetics are summarised in Fig. 1B and given by the following equations:

(2) $$\frac{d[F_{A}]}{\mathrm{dt}}=k_{b}(F_{\max}-[F_{A}]-[V_{A}]){\left[{\mathrm{Ca}}^{2+}\right]}_{\mathrm{syn}}^{4}-k_{u}[F_{A}]-k_{1}[F_{A}][V]+k_{2}[V_{A}],$$

(3) $$\frac{d[V_{A}]}{\mathrm{dt}}=k_{1}[F_{A}][V]-(k_{2}+k_{3})[V_{A}],$$

(4) $$\frac{d[\mathrm{NA}]}{\mathrm{dt}}={\mathrm{Nk}}_{3}[V_{A}]-k_{h}[\mathrm{NA}].$$

Here, calcium ions are assumed to reversibly bind to a fusion protein *F*. Four calcium ions bind to this protein at a rate *k*_*b*_, changing it to its activated state *F*_*A*_. The reverse process has an unbinding rate *k*_*u*_. Destexhe et al. (1994) simplified this system by assuming that there exists an inexhaustible pool of pre-docked vesicles *V*, with concentration [V], that are ready for activation. The activation of a vesicle *V* by an activated fusion protein *F*_*A*_ has forward and backward rate-constants of *k*_1_ and *k*_2_, respectively. The activated vesicle, *V*_*A*_, is then able to fuse to the membrane of the synaptic terminal, and subsequently release its contents into the neuromuscular junction. An activated vesicle *V*_*A*_ releases *N* molecules of NA into the synaptic junction at a rate *k*_3_. [NA] is the concentration of the released ligand NA. The released transmitter is removed from the synaptic cleft by diffusion, degradation and reuptake, all of which are incorporated into the clearance rate-constant *k*_*h*_.

### 2.3 G-protein activation in smooth muscle cells

Released NA diffuses across the neuromuscular junction, and binds to *α*_1_-adrenoreceptors (*α*_1_R) on the SMC membrane (Fig. 1C_1_). This is a G ${}_{q/11}$-protein coupled receptor. On binding NA the *α*_1_R undergoes a conformation change, activating the intracellularly coupled G-protein ($G_{q/11}$), which detaches from the receptor, dissociating into subunits. This detached/activated G-protein triggers a second messenger cascade within the SMC, leading to a contractile response.

A model of the activation, phosphorylation and internalisation of a G-protein coupled receptor has previously been described by Bennett and co-workers and is used here for modelling intra-SMC signalling (Lemon et al., 2003; Bennett et al., 2005). The specific equations model the activation of an *α*_1_R following binding of NA, and the subsequent decoupling of a G-protein:

(5) $$\frac{d[R^{S}]}{\mathrm{dt}}=k_{r}\zeta [R_{T}]-(k_{r}+\frac{k_{p}[\mathrm{NA}]}{K_{1}+[\mathrm{NA}]})[R^{S}]-k_{r}[R_{P}^{S}]$$

(6) $$\frac{d[R_{P}^{S}]}{\mathrm{dt}}=[\mathrm{NA}](\frac{k_{p}[R^{S}]}{K_{1}+[\mathrm{NA}]}+\frac{k_{e}[R_{P}^{S}]}{K_{2}+[\mathrm{NA}]})$$

Here, $\left[R^{S}\right]$ and $\left[R_{P}^{S}\right]$ are the numbers of unphosphorylated and phosphorylated surface receptors, respectively. These equations assume that the binding of the ligand NA is rapid; the implications of which were formalised by Wagner and Keizer (1994). *K*_1_ and *K*_2_ are dissociation constants and $\left[R_{T}\right]$ is the total number of receptors. The parameter ζ=0.85 takes into account the assumption that only 85% of receptors will be participating in G-protein signalling. The parameters *k*_*p*_, *k*_*e*_, *k*_*r*_ are the rates of receptor phosphorylation, internalisation and recycling, respectively. For a full derivation of the receptor equations, see Lemon et al. (2003).

Only the unphosphorylated surface receptors $\left[R^{S}\right]$ are able to bind NA and activate the G-protein cascade that leads to SMC contraction. As formalised by Lemon et al. (2003), the concentration of activated G-protein [G] satisfies an equation of the form

(7) $$\frac{d[G]}{\mathrm{dt}}=k_{a}(\delta +p_{r}([R^{S}],[\mathrm{NA}]))([G_{T}]-[G])-k_{d}[G].$$

Here, the rate-constants *k*_*a*_ and *k*_*d*_ are of G-protein activation and deactivation, respectively, and $\left[G_{T}\right]$ is the total number of G-protein molecules. The constant *δ* represents the background contribution of NA-unbound, unphosphorylated surface receptors to G-protein activation. The function *p*_*r*_ is the main contribution to G-protein activity, representing the activation of G-protein molecules by NA-bound unphosphorylated surface receptors. The form of this function is given by

(8) $$p_{r}([R^{S}],[\mathrm{NA}])=\frac{\left[\mathrm{NA}][R^{S}\right]}{\zeta [R_{T}](K_{1}+[\mathrm{NA}])}.$$

### 2.4 IP_3_-induced Ca^2+^ release

The activated G-protein triggers a protein cascade, resulting in the release of Ca^2+^ from intracellular stores within the SMC. The kinetics of this process are shown in Fig. 1C_1_ and can be described as follows. An inactive G-protein G·GDP can bind to a NA-bound *α*_1_R. Once NA binds to the *α*_1_R then guanosine diphosphate (GDP) is exchanged for guanosine-5 ${}^{\prime }$-triphosphate (*GTP*). The G-protein dissociates from the receptor to form the complex G·GTP. Once dissociated, the G-protein disassembles into subunits. The active subunit is ($G_{\alpha }\cdot \mathrm{GTP}$). This activated G-protein is able to bind to phospholipase C (PLC). This G-protein—PLC complex can then hydrolyse PIP_2_ (that resides in the SMC membrane) to inositol 1,4,5-trisphosphate (IP_3_. This process is catalysed by Ca^2+^.

We have adopted the model of this G-protein cascade derived by Bennett and co-workers (Lemon et al., 2003; Bennett et al., 2005). Here, the active G-protein complex $G_{\alpha }\cdot \mathrm{GTP}$ is denoted simply by G. The rate of hydrolysis of PIP_2_ is $r_{h}[{\mathrm{PIP}}_{2}]$, where [${\mathrm{PIP}}_{2}$] is the total number of PIP_2_ molecules in the cell membrane and $r_{h}$ is the rate at which one molecule is hydrolysed into IP_3_, given by

(9) $$r_{h}=\alpha (\frac{{\left[{\mathrm{Ca}}^{2+}\right]}_{\mathrm{SMC}}}{K_{C}+{\left[{\mathrm{Ca}}^{2+}\right]}_{\mathrm{SMC}}})[G].$$

Here, *α* is an ‘effective signal gain parameter׳, and $K_{C}$ is the dissociation constant for the Ca^2+^ binding site on the PLC molecule. ${\left[{\mathrm{Ca}}^{2+}\right]}_{\mathrm{SMC}}$ is the intra-SMC concentration of Ca^2+^.

The rate of change of IP_3_ is determined by the rate at which PIP_2_ is hydrolysed and IP_3_ is degraded, replenishing PIP_2_ levels in the cell membrane. The means by which PIP_2_ is replenished can be summarised by the degradation of IP_3_ to an intermediate phospholipid, which is then phosphorylated and returned to the cell surface. These two processes can be modelled as follows:

(10) $$\frac{d[{\mathrm{IP}}_{3}]}{\mathrm{dt}}=r_{h}\gamma^{-1}[{\mathrm{PIP}}_{2}]-k_{\deg}[{\mathrm{IP}}_{3}]$$

(11) $$\frac{d[{\mathrm{PIP}}_{2}]}{\mathrm{dt}}=-(r_{h}+r_{r})[{\mathrm{PIP}}_{2}]-r_{r}\gamma [{\mathrm{IP}}_{3}]+r_{r}[{\left({\mathrm{PIP}}_{2}\right)}_{T}]$$

As in Lemon et al. (2003) and Bennett et al. (2005), we require IP_3_ as a concentration for subsequent equations. Therefore, [${\mathrm{IP}}_{3}$] is the concentration of IP_3_, [${\mathrm{PIP}}_{2}$] is the number of PIP_2_ molecules and the factor *γ* (Avogadro׳s number, *N*_*a*_×cell volume, *C*_*V*_) converts concentration into particle number. [${\left({\mathrm{PIP}}_{2}\right)}_{T}$] is the total pool of phospholipid to which IP_3_ is returned. The replenishment of PIP_2_ levels in the membrane assumes degradation of IP_3_ to the intermediate phospholipid at a rate $k_{\deg}$, and then phosphorylation of this phospholipid to PIP_2_ at a rate $r_{r}$.

The IP_3_ molecule activates IP_3_ receptors (IP_3_R) on the membrane of the sarcoplasmic reticulum (SR) within the SMC. Activation of this receptor causes the release of calcium into the cytosol of the SMC, increasing the intra-SMC concentration of Ca^2+^, ${\left[{\mathrm{Ca}}^{2+}\right]}_{\mathrm{SMC}}$. A two-variable model describing the calcium fluxes across an SR membrane was first described by Li and Rinzel (1994), and employed by Fink et al. (1999). The ${\left[{\mathrm{Ca}}^{2+}\right]}_{\mathrm{SMC}}$ dynamics are governed by fluxes across the SR membrane from a leak flux *J*_*leak*_, a calcium pump flux *J*_*pump*_, and a receptor flux generated by IP_3_ channels *J*_*IP*3_. Released calcium is buffered at a constant rate *β*, giving

(12) $$\frac{d{\left[{\mathrm{Ca}}^{2+}\right]}_{\mathrm{SMC}}}{\mathrm{dt}}=\beta (J_{\mathrm{IP}3}-J_{\mathrm{pump}}+J_{\mathrm{leak}})$$

(13) $$\frac{\mathrm{dh}}{\mathrm{dt}}=k_{\mathrm{on}}(K_{\mathrm{inh}}-({\left[{\mathrm{Ca}}^{2+}\right]}_{\mathrm{SMC}}+K_{\mathrm{inh}})h).$$

The additional variable *h* is a pseudo-gating variable for the IP_3_R channels. The three fluxes are given by

$$J_{\mathrm{IP}3}=J_{\max}{\left[(\frac{\left[{\mathrm{IP}}_{3}\right]}{[{\mathrm{IP}}_{3}]+K_{I}})(\frac{{\left[{\mathrm{Ca}}^{2+}\right]}_{\mathrm{SMC}}}{{\left[{\mathrm{Ca}}^{2+}\right]}_{\mathrm{SMC}}+K_{\mathrm{act}}})h\right]}^{3}[1-\frac{{\left[{\mathrm{Ca}}^{2+}\right]}_{\mathrm{SMC}}}{{\left[{\mathrm{Ca}}^{2+}\right]}_{\mathrm{SR}}}]J_{\mathrm{leak}}=P_{L}(\frac{{\left[{\mathrm{Ca}}^{2+}\right]}_{\mathrm{SMC}}}{{\left[{\mathrm{Ca}}^{2+}\right]}_{\mathrm{SR}}})J_{\mathrm{pump}}=V_{\max}\frac{{\left[{\mathrm{Ca}}^{2+}\right]}_{\mathrm{SMC}}^{2}}{{\left[{\mathrm{Ca}}^{2+}\right]}_{\mathrm{SMC}}^{2}+K_{p}}.$$

### 2.5 Smooth muscle cell contraction

The basis of contraction of an SMC involves the binding of phosphorylated myosin to actin, forming a ‘cross-bridge’ (Fig. 1C_2_). The kinetic model we use to describe this process is again that used by Bennett et al. (2005), which is a reduction of the original model by Hai and Murphy (1988).

In the model, the states of actin A and myosin M are considered. These can be phosphorylated (_*p*_), and bound to each other. Free Ca^2+^ molecules are able to trigger a process leading to phosphorylation of the myosin head (M_*P*_), cocking it. This process happens at a rate k^1=k^m[Ca2+]4. Phosphorylation of M is reversible, with an unbinding rate k^2. Once the myosin head is cocked it is able to attach to actin and generate a contractile force. The cocked head ‘latches’ to the actin filament at a rate constant k^3, with a de-latching rate k^4. The latching of a cocked myosin head M_*P*_ to an actin filament forms a cross-bridge AM_*P*_. The latched and cocked cross-bridge may now perform a contractile power-stroke, using the energy stored in the myosin head as adenosine diphosphate (ADP).

By assuming that there is a total, conserved concentration of myosin, $\left[M_{T}]=[M]+[M_{P}]+[{\mathrm{AM}}_{P}\right]$, it is possible to derive the simplified kinetics

(14) d[M]dt=k^2([MT]−[AMP])−(k^m[Ca2+]SMC4+k^2)[M]

(15) d[AMP]dt=k^3([MT]−[M])−(k^3+k^4)[AMP]

Finally, the contractile force produced by the SMC is assumed to be proportional to the concentration $\left[{\mathrm{AM}}_{P}\right]$ of cross-bridges.

## 3 Model adaptation and simulation

Here we outline the parameter values of the model, any adaptations we have made to the equations or parameters, and details of simulations.

### 3.1 Noradrenaline release kinetic parameters

The parameter values for NA release kinetics are given in Table 1, together with references supporting the chosen value. The parameter values for the NA release model—Eqs. (1)–(4)—are as in the original model of transmitter release by Destexhe et al. (1994). The only deviation is the value of the parameter *k*_*h*_, which encapsulates the rate of clearance, degradation and reuptake of the released transmitter. Carbon fibre studies of NA release onto the rat tail artery, have revealed that it is cleared by neuronal reuptake and diffusion, while extraneuronal uptake is negligible (Stjarne et al., 1994). Since diffusion of NA is known to be restricted, due to the dense plexus of connective tissue surrounding release sites, we set $k_{h}=0.003\;{\mathrm{ms}}^{-1}$ (Gonon et al., 1993b). We found that with this parameter value, stimulation of the SPGN model with a single pulse, resulted in a clearance of released NA that took ≈1.5s, fitting carbon-fibre observations (Stjarne et al., 1994; Stjarne, 2000). Furthermore, stimulation of the model with action potentials at 12 Hz produced a NA concentration of 100 nM (see Fig. 4). This fit experimental data of Gonon et al. (1993a), which found the concentration to reach 120 nM.

### 3.2 G-protein cascade in smooth muscle cells

The parameters of Eqs. (5)–(11) are given in Tables 1 and 2. All parameter values are as in the original model of a G-protein cascade following activation of a P_2_Y_2_ purinergic receptor (Lemon et al., 2003; Bennett et al., 2005). However, we have used a timescale argument to reduce Eqs. (5)–(13). This also made the system less numerically stiff. Examination of the time constants reveals that receptor and G-protein dynamics occur on a timescale that is at least one order of magnitude faster than all other dynamic features of the model. Therefore to make the model less numerically stiff, and to reduce the dimension of the model we suppose that the receptor and G-protein dynamics are instantaneous and therefore these concentrations are replaced by their steady-state values. In particular, for a given noradrenaline concentration [NA], Eqs. (5)–(7) can be replaced by the linear system of algebraic equations

(16) $$(\begin{array}{cc} k_{r}+\frac{k_{p}[\mathrm{NA}]}{K_{1}+[\mathrm{NA}]} & k_{r}\\ \frac{k_{p}[\mathrm{NA}]}{K_{1}+[\mathrm{NA}]} & -\frac{\mathrm{ke}[\mathrm{NA}]}{K_{2}+[\mathrm{NA}]} \end{array})(\begin{array}{c} \overset{¯}{\left[R^{S}\right]}\\ \overset{¯}{\left[R_{P}^{S}\right]} \end{array})=(\begin{array}{c} k_{r}\zeta [R_{T}]\\ 0 \end{array}),$$

where

(17) $$\underset{t\to \infty }{\lim}[R^{S}]=\overset{¯}{\left[R^{S}\right]}$$

(18) $$\underset{t\to \infty }{\lim}[R_{P}^{S}]=\overset{¯}{\left[R_{P}^{S}\right]}$$

We can then determine the steady state of activated G-protein via

(19) $$\overset{¯}{\left[G\right]}=\frac{\left[G_{T}]k_{a}(\delta +p_{r}(\overset{¯}{\left[R^{S}\right]},[\mathrm{NA}])\right)}{k_{d}+k_{a}(\delta +p_{r}(\overset{¯}{\left[R^{S}\right]},[\mathrm{NA}])},$$

**Fig. 2:**
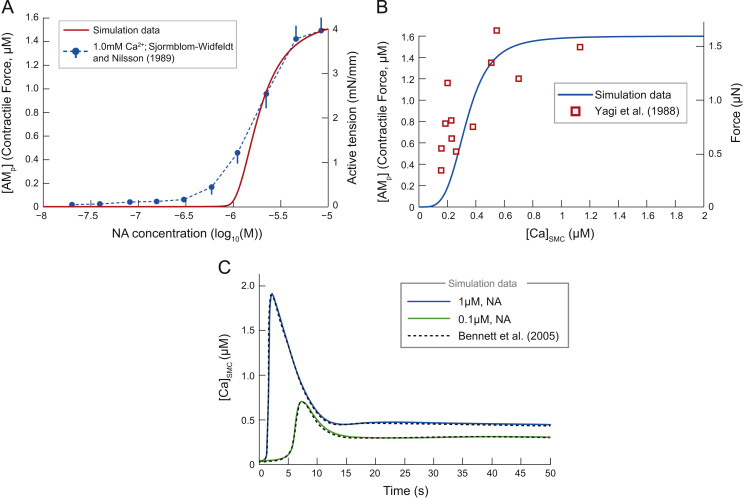
Response of model to application of exogenous noradrenaline and endogenous calcium. (A) $\left[{\mathrm{AM}}_{P}\right]$ response to constant application of [NA]. Simulation data (solid) exhibits a steeper relation than the experimental data (broken lines) of Sjoblom-Widfeldt and Nilsson (1989) for the tensile response of small mesenteric arteries. Experimental data points (±SEM) are conducted in the presence of extracellular calcium concentration 1.0 mM. Note the fitting of the model to the experimental data for high [NA], which diverges when [NA]<1.8μM. (B) The force developed by our SMC model as a function of ${\left[{\mathrm{Ca}}^{2+}\right]}_{\mathrm{SMC}}$. The concentration of actin bound to phosphorylated myosin $\left[{\mathrm{AM}}_{P}\right]$ in the model, followed a sigmoid relationship. Experimental observations from Yagi et al. (1988) show that the $\left[{\mathrm{AM}}_{P}\right]$ closely resembles the force in μN. (C) The response of ${\left[{\mathrm{Ca}}^{2+}\right]}_{\mathrm{SMC}}$ to exogenous application of [NA]=1μM (blue) and 0.1μM (green). The delayed, transient rise, followed by a steady-state plateau fits simulations of Bennett et al. (2005) (dashed), and therefore similarly fits experimental data of Li et al. (1993). These data support our quasi-steady state assumption for the receptor dynamics. (For interpretation of the references to color in this figure caption, the reader is referred to the web version of this article.)

This steady-state $\overset{¯}{\left[G\right]}$ can then be used in Eqs. (9)–(11) in place of the dynamic variable [G]. Fig. 2C validates this steady-state assumption. The intracellular calcium concentration ${\left[{\mathrm{Ca}}^{2+}\right]}_{\mathrm{SMC}}$ in response to [NA] concentrations of 0.1μM and 1μM closely matches those of the original system by Bennett et al. (2005).

### 3.3 IP_3_-induced Ca^2+^ release

The parameters of Eq. (12) are given in Table 2. Their values are taken from a model of Ca^2+^ dynamics following IP_3_ uncaging in A7r5 cells (a cultured rat SMC line from the thoracic aorta) (Fink et al., 1999). This is the same parameter set as used in Bennett et al. (2005).

### 3.4 Cross-bridge formation in smooth muscle cells

The parameter values for Eqs. (14) and (15) are given in Table 2. The parameters values are as in Bennett et al. (2005) except for the total concentration of myosin, which was set to $[M_{T}]=2.3$. This gives a correspondence between the units of $\left[{\mathrm{AM}}_{P}\right]$ and the contractile force generated by the SMC seen experimentally by Yagi et al. (1988) (see Fig. 2B). The variable $\left[{\mathrm{AM}}_{P}\right]$ is therefore referred to as the contractile force produced by the SMC model, with units of μN.

### 3.5 Simulation methodology

Simulations of our single-cell model of an SPGN were performed with the programme NEURON (version 7.3; Carnevale and Hines, 2006) using a 12.5μs time step. The response *I*_*Ca*_ of the SPGN model was recorded at the distal end of the axon and imported into MATLAB (version 6.1) within which the rest of the model was run.

The *I*_*Ca*_ time series was used to force Eq. (1), which then initiates NA release via Eqs. (2)–(4). The released NA was used to drive the steady-state Eqs. (16) and (19), describing *α*_1_R and subsequent G-protein activation. This concentration of activated G-protein triggered IP_3_ release, governed by Eqs. (10) and (11). The increase in $\left[{\mathrm{IP}}_{3}\right]$ increases ${\left[{\mathrm{Ca}}^{2+}\right]}_{\mathrm{SMC}}$ as formalised by (12) and (13), which in turn triggers the contractile dynamics, given by (14) and (15).

Simulations of our model were performed on a two dual-core Opteron processors 8 GB RAM node, using the computational facilities of the Advanced Computing Research Centre, University of Bristol, UK (http://www.bris.ac.uk/acrc/).

## 4 Results

### 4.1 Model validation

To test the correctness of our adaptation to Bennett et al.׳s (2005) model, different components of the model were directly simulated and compared with experimental data. First, the model was directly stimulated with fixed concentrations of NA to mimic exogenous bath application of NA and other *α*_1_R agonists (Fig. 2A). The SMC contractile response followed a log sigmoidal relationship with [NA]. At 1μM ($\log_{10}(M)=-6$) the response begins to rise, with an ${\mathrm{ED}}_{50}=1.47\;\mu M$ ($\log_{10}(M)=-5.83$). At ≈10μM ($\log_{10}(M)=-5$) a maximal contraction is reached. Experimental data of Sjoblom-Widfeldt and Nilsson (1989) is also shown. In these experiments, the active tension of mesenteric arteries in male Wistar rats in response to NA application, in the presence of extracellular calcium concentration 1.0 mM, was measured. The *ED*_50_ of these experimental data was 1.48μM, providing a good fit with our simulation data. In particular the model provides an excellent fit to the top part of the experimental response curves, but begins to rise at a higher NA concentration and with a steeper relationship to $\left[{\mathrm{AM}}_{P}\right]$. Note however that the simulations are for a single SMC, whereas the experimental data is for an arterial response (see Section 5 for further discussion).

In addition, we explored the characteristics of the actin–myosin model (Eqs. (14) and (15)) by driving it with a fixed level of ${\left[{\mathrm{Ca}}^{2+}\right]}_{\mathrm{SMC}}$ held at concentrations between 0–2μM (Fig. 2B). Note how the simulated steady-state curve fits the experimental data of Yagi et al. (1988). Both the experimental and simulated response begin to rise at ${\left[{\mathrm{Ca}}^{2+}\right]}_{\mathrm{SMC}}=0.1\;\mu M$ and reach a maximum at ${\left[{\mathrm{Ca}}^{2+}\right]}_{\mathrm{SMC}}=1\;\mu M$.

Finally, the response of ${\left[{\mathrm{Ca}}^{2+}\right]}_{\mathrm{SMC}}$ to exogenous application of NA was recorded (Fig. 2C). Fig. 2C shows the dynamic ${\left[{\mathrm{Ca}}^{2+}\right]}_{\mathrm{SMC}}$ response to [NA]=0.1μM and 1μM. Note how the calcium concentration experiences a delayed transient peak, followed by a steady-state plateau, as in experimental observations (Li et al., 1993). These simulations also fit those of Bennett et al. (2005), justifying our steady-state approximation of the G-protein and receptor dynamics given in Eqs. (16) and (19).

**Fig. 3:**
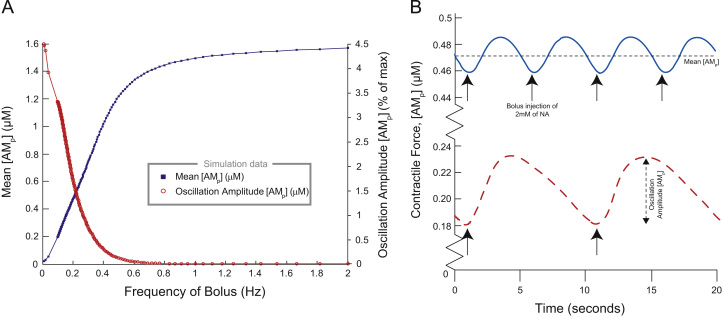
Contractile response of SMC model to periodic stimulation. The SMC model was stimulated periodically for 300 ms with a 2 nM bolus of NA, at various frequencies (0.01–2 Hz). (A) Across one period of stimulation, the mean $\left[{\mathrm{AM}}_{P}\right]$ increased with frequency, whereas the amplitude of oscillations in $\left[{\mathrm{AM}}_{P}\right]$ (as a % of the maximum contractile force) decreased. Above 0.4 Hz, oscillations in $\left[{\mathrm{AM}}_{P}\right]$ were negligible (<1% of max). (B) Contractile force $\left[{\mathrm{AM}}_{P}\right]$ to stimulation at 0.1 Hz (red, dashed) and 0.2 Hz (blue). (For interpretation of the references to color in this figure caption, the reader is referred to the web version of this article.)

A further test to the model was to expose it periodically to a 300 ms bolus of [NA] (2 mM) at frequencies 0.01–2 Hz (Fig. 3). The steady-state mean $\left[{\mathrm{AM}}_{P}\right]$ and amplitude of any oscillations in $\left[{\mathrm{AM}}_{P}\right]$ are plotted against frequency in Fig. 3A. At high frequencies, the model cell contracted and was unable to relax before the next bolus of NA occurred; the magnitude of oscillations was seen to decrease quickly with increasing stimulation frequency. Such a tonic contraction occurred at stimulation frequencies above 0.4 Hz. At higher stimulation frequencies, the mean contractile force is greater, but the oscillation amplitude reduced (Fig. 3B). These results match those found experimentally in vascular SMCs isolated from the aorta of rats (Julien et al., 2001), where SMCs only produced oscillatory contractions (in response to repetitive boluses of the *α*_1_R agonist phenylephrine) when the stimulation frequency was ≤0.3Hz.

### 4.2 Tonic stimulation of model

To determine the response of the SMC to tonic firing, the SPGN model was driven with a train of current pulses (2 nA×2 ms) at a range of frequencies (1–25 Hz). These frequencies are consistent with those seen in muscle vasoconstrictor (MVC) sympathetic preganglionic neurones of normotensive (Wistar-Kyoto, WKY) rats (Malpas, 1998; Stalbovskiy et al., 2014; Briant et al., 2014).

**Fig. 4:**
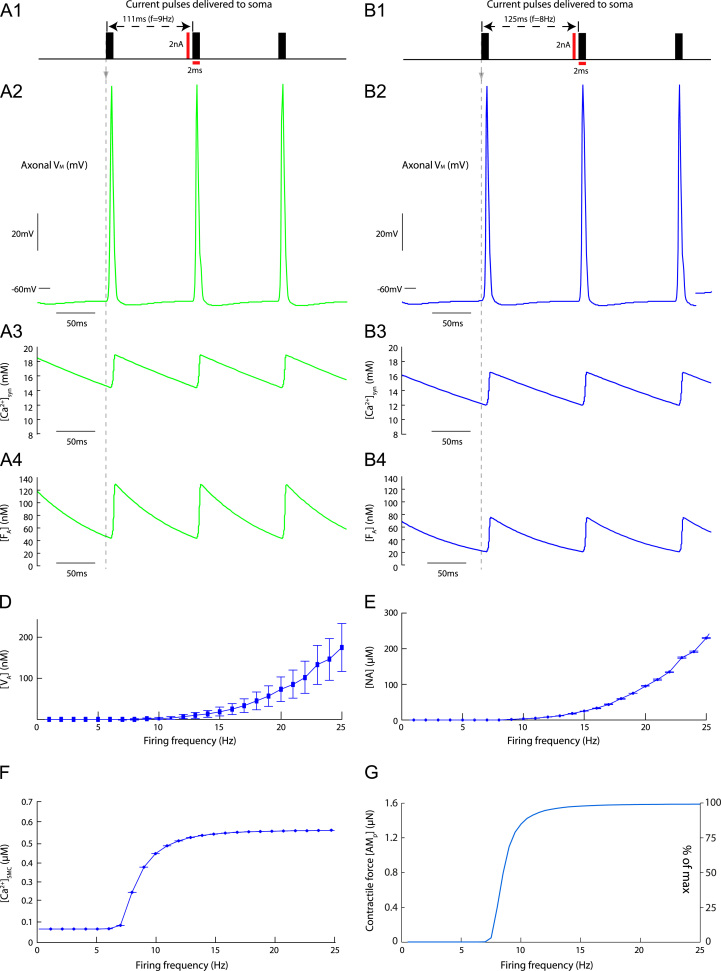
Response of the model to tonic stimulation. Response of the model to tonic stimulation. Tonic stimulation at 9 Hz (A) and 8 Hz (B). (A_1_, B_1_) The pulses played into the SPGN model, to generate the said firing frequencies. (A_2_, B_2_) The action potential waveforms in the axon terminal, in response to these pulses. (A_3_, B_3_) ${\left[{\mathrm{Ca}}^{2+}\right]}_{\mathrm{syn}}$ increases, driven by these action potentials. Summation is linear across the frequencies. (A_4_, B_4_) $\left[F_{A}\right]$ summates nonlinearly, with greater peaks in activation at high-frequency. (D) $\left[V_{A}\right]$ increases with stimulus/firing frequency. Error bars are the maximum and minimum of oscillations in this concentration due to the periodic stimulus. (E) [NA] increases with firing frequency. Oscillations are of negligible (<0.1 nM) amplitude. (F) Released NA leads to increases in ${\left[{\mathrm{Ca}}^{2+}\right]}_{\mathrm{SMC}}$, due to *α*_1_R and G-protein activation. This increases in a sigmoidal fashion at ≈7Hz. (G) Contractile response of SMC model to tonic stimulation of SPGN model. As firing frequency increases, the contractile force $\left[{\mathrm{AM}}_{P}\right]$ increases sigmoidally. Below 7 Hz, the SMC is unresponsive to tonic stimulation.

Stimulating the model with current pulses at 9 Hz (Fig. 4A) and 8 Hz (Fig. 4B) caused contraction of the smooth muscle cell model. Each pulse (Fig. 4A_1_ and B_1_) triggered an action potential in the soma, which propagated down the axon of the SPGN model (Fig. 4A_2_ and B_2_). L- and N-type calcium channels activated causing an increase in ${\left[{\mathrm{Ca}}^{2+}\right]}_{\mathrm{syn}}$ (Fig. 4A_3_ and B_3_). The concentration of activated fusion protein, $\left[F_{A}\right]$ was found to increase with firing frequency (Fig. 4A_4_ and B_4_). At higher stimulation frequencies, the amplitude of $\left[F_{A}\right]$ oscillations increases. Because F_*A*_ binds to vesicles V, activating them for release, it was consequently found that both the mean concentration (data point) and pulse-to-pulse amplitude (error bars) of activated vesicles $\left[V_{A}\right]$ increased with stimulation frequency (Fig. 4D). Similarly, driven by the nonlinearity in $\left[F_{A}\right]$ it was found that both the mean and the oscillatory response of $\left[V_{A}\right]$ to spikes in ${\left[{\mathrm{Ca}}^{2+}\right]}_{\mathrm{syn}}$ increase non-linearly with firing frequency. Hence, as activated vesicles dock and NA is released into the synaptic cleft, it is found that [NA] increases nonlinearly with firing frequency, but is proportional to the increase in $\left[V_{A}\right]$ (Fig. 4E). Notice how the oscillations in [NA] were found to have negligible amplitude at high frequency, being <9μM in magnitude.

The subsequent G-protein cascade causes a rise in the post-junctional concentration of calcium, ${\left[{\mathrm{Ca}}^{2+}\right]}_{\mathrm{SMC}}$, as shown in Fig. 4F. At low frequency (≤7Hz) ${\left[{\mathrm{Ca}}^{2+}\right]}_{\mathrm{SMC}}$ is about 0.06μM, which begins to rise non-linearly at about 7 Hz, reaching a plateau of 0.6μM at approximately 15 Hz. The steady-state amplitudes of ${\left[{\mathrm{Ca}}^{2+}\right]}_{\mathrm{SMC}}$ oscillations can be seen to be negligible (<0.01μM).

The steady-state contractile force $\left[{\mathrm{AM}}_{P}\right]$ in response to tonic SPGN stimulation is shown in Fig. 4G. This sigmoidal relationship began rising at 7 Hz and peaked at $[{\mathrm{AM}}_{P}]=1.6\;\mu N$ by 12 Hz. For tonic stimulation at <7Hz, the contractile response of the SMC was minimal (<1%). The simulation data had an ${\mathrm{ED}}_{50}=7.3\;\mathrm{Hz}$ which is a good fit to experimental data, where the *ED*_50_ of mesenteric arteries is between 6 and 8 Hz (Nilsson et al., 1985; Sjoblom-Widfeldt and Nilsson, 1989).

### 4.3 Paired-pulse stimulation of the model

To understand the relationship between firing frequency and NA release, pairs of action potentials were generated in the SPGN, with varying inter-pulse intervals (IPIs) of 40–2000 ms. We were particular interested in the response to paired pulses separated by <100ms, as bursting in sympathetic efferents can reach instantaneous frequencies of >10Hz.

**Fig. 5:**
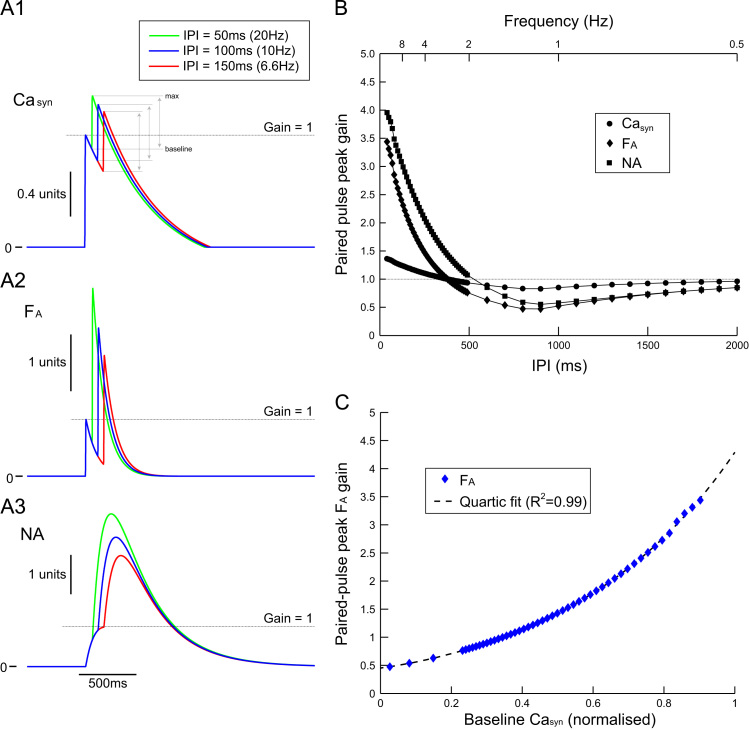
Paired-pulse stimulation of noradrenaline release. Paired-pulse stimulation of noradrenaline release. The soma of the SPGN model was driven with a pair of pulses (2 ms×2 nA) at a fixed inter-pulse interval (IPI) ranging from 40–2000 ms. These pulses drove NA release via Eqs. (1)–(5). The variables of these equations were normalised by the single-pulse response, to measure the gain in the second pulse as a function of IPI. (A) The response of pre-synaptic calcium (Ca_*syn*_, A_1_), activated fusion protein (F_*A*_, A_2_) and noradrenaline (NA, A_3_) to paired-pulse stimulation at 50 (green), 100 (blue) and 150 ms (red). The gain in the second pulse in Ca_*syn*_ was similar across the IPIs. The gain in F_*A*_ increased as IPI decreased. (B) Paired-pulse peak gain in Ca_*syn*_ (circle), F_*A*_ (diamond) and NA (square) as a function of IPI. Gain was measured as the maximal height reached in response to the second response, as a proportion of the height of the first response (gain=1). Note that at high-frequency (IPI<100ms) the gain in Ca_*syn*_ is small (1.3–1.4) and a shallow function of IPI. Contrarily, the gain in F_*A*_ is large (2.5–3.5) and a steep function of IPI. This drives greater release of NA, which exhibits an even greater gain (3–4) due to slow reuptake. (C) The relationship between the gain in activation of the fusion protein and the baseline pre-synaptic calcium level at the time of arrival of the second action potential is quartic. (For interpretation of the references to color in this figure caption, the reader is referred to the web version of this article.)

The paired action potentials propagated down the axon to drive the pre-synaptic calcium concentration and therefore trigger NA release from the axon terminal (Fig. 5). The peak response of pre-synaptic calcium (Ca_*syn*_—normalised to the response to first pulse) to the second pulse, was not greatly influenced by the IPI (Fig. 5A_1_ and B). In particular, the amplitude of the Ca_*syn*_ response to the second pulse was similar for 50 ms, 100 ms and 150 ms IPIs (Fig. 5A_1_). The peak gain in Ca_*syn*_ increased non-linearly as IPI is reduced, but this relationship was relatively gentle (compared to other variables; Fig. 5B). Fusion proteins (F) were activated (F_*A*_) by the increase in pre-synaptic calcium. The response of F_*A*_ to the second pulse (normalised by the response to the first pulse) was greatly increased as the IPI was reduced from 150 ms to 50 ms (Fig. 5A_2_). Because of this, the gain in activated fusion proteins in response to the second pulse, could reach 2.5–3.5 times that of the first pulse for small (<100ms) IPIs (Fig. 5B), much greater than the paired-pulse gain of Ca_*syn*_. The gain in activation of vesicles was similar to that of F_*A*_, being linearly related.

The paired-pulse gain for NA was also relatively large (Fig. 5A_3_), reaching 3–4 times that of the first pulse for short IPIs (<100ms; Fig. 5B). This was driven by the paired-pulse gain in F_*A*_ and the slow time-course of decay of NA.

The paired-pulse gain in F_*A*_ was plotted as a function of the “baseline” (see Fig. 5A_1_) level of Ca_*syn*_ (Fig. 5C). The data show that the paired-pulse gain in F_*A*_ is a function of the Ca_*syn*_ level at the time of arrival of the second spike; when the baseline level of Ca_*syn*_ approaches 1—tantamount to the IPI being reduce—the paired-pulse gain in F_*A*_ can reach 4. These data are fit with a quartic polynomial. Therefore, the large paired-pulse gain in fusion protein activation and NA release for small IPI is because F_*A*_ is a quartic function of the basal level of Ca_*syn*_.

### 4.4 Spike-train driven model

**Fig. 6:**
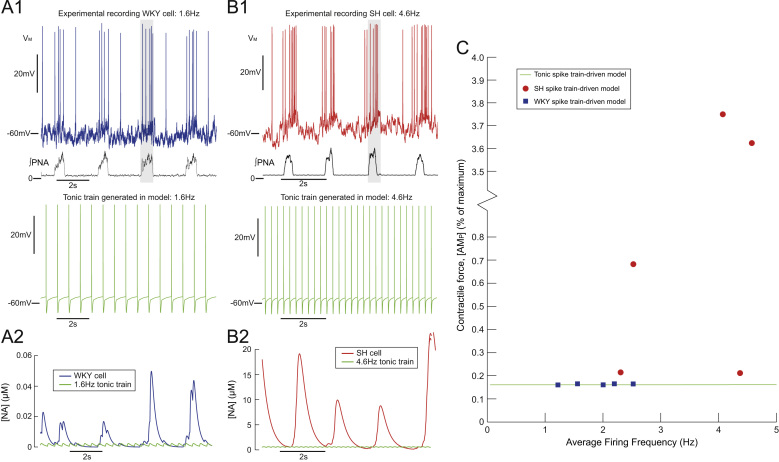
Spike train model produces greater release of NA than tonic drive model. Spike train model produces greater release of NA than tonic drive model. Experimental recordings of membrane potential (*V*_*M*_) in an MVC_*like*_ sympathetic preganglionic neurone in a normotensive (WKY; (A)) and spontaneously hypertensive (SH; (B)) (Briant et al., 2014) were used to drive contraction in the SMC model. The WKY cell fired at an average frequency of 1.6 Hz and the SH cell at 4.6 Hz. Both recordings exhibit respiratory modulation, with increases in firing frequency entrained to PNA (lower trace; shaded region). The response of the model to these patterns was compared to tonic stimulation at the same average firing frequency. The NA released in response to the bursting spike train, was greater than tonic, for both the WKY (A_*2*_) and SH (B_*2*_) recording. The release of NA was entrained to respiration. The contractile response was greater in response to the bursting spike trains, but was greatest in response to the SH spike train. (C) Steady-state contractile response of SMC model (% of maximum) to n=5 WKY spike trains (square) and n=5 SH spike trains (circle). The response to tonic stimulation at the same average firing frequency is also shown (dashed line).

The model was driven with experimentally recorded spike trains and for comparison tonic trains at the same average firing frequency (Fig. 6). Membrane potential was recorded *in situ* from muscle vasoconstrictor-like sympathetic preganglionic neurones in normotensive (WKY) and spontaneously hypertensive rats by whole-cell patch clamping techniques (Stalbovskiy et al., 2014; Briant et al., 2014). The spike trains from cell recordings of a WKY and SH rats are shown in Fig. 6A_1_ and B_1_, respectively. The discharge exhibited the typical respiratory modulation of firing, with an increase in firing occurring during inspiration (as marked by phrenic nerve activity (PNA); see Fig. 6A_1_ and B_1_ lower traces and shaded regions). Note that the average firing frequency of the spike trains was subthreshold for a SMC contractile response, being 1.6 Hz for the WKY and 4.6 Hz for the SH rat (see Fig. 4G). Note also that the SH spike train exhibits increased respiratory modulated bursting, with spiking during PNA that peaks at 12 Hz (vs 5 Hz in the WKY cell). This is a characteristic of sympathetic activity in hypertension (Simms et al., 2009; Briant et al., 2014). The raw voltage-traces were converted into spike trains to drive the SPGN model—at each action potential a pulse (2 ms×2 nA) was played into the soma, to trigger an action potential in the model. The response of the SMC model to these inputs was compared to the response to tonic inputs of the same average firing frequency.

Each action potential in the spike train triggered a transient calcium current in the distal end of the axon, driving fusion protein activation and NA release the neuromuscular junction (Fig. 6A_2_ and B_2_). The amount of NA released was found to increase phasically with PNA, due to bursts of action potentials occurring during inspiration. The concentration of NA released was greater than that produced by tonic stimulation at the same average firing frequency. The SH spike train triggered the greatest release of NA (note the difference in axis) which was entrained to PNA.

The SPGN model was driven by 10 spike trains recorded from WKY (n=5) and SH (n=5) rats in Briant et al. (2014) (Fig. 6C). Each spike train consisted of 5–10 respiratory cycles of spiking data. Spiking increased during PNA (inspiration). The WKY spike trains had low average firing frequencies (<2.5Hz) and were characterised by bursts of 3–5 spikes during the inspiration period. The contractile force produced by the WKY spike trains was small, being similar to the force produced by tonic stimulation at the same average firing frequency (≈0.1% of the maximal force available). The SH spike trains had larger average firing frequencies (2.3–4.6 Hz), and had characteristic respiratory bursts of larger amplitude (more spikes per burst). The contractile response to the SH spike trains was 0.2–3.8% of the force available. These larger contractions were also greater than the contractions produce by tonic stimulation at the same average firing frequency. Two SH spike trains produced small contractions (≈0.2% of maximum), that were still greater than tonic and WKY stimulation. These data show that SH spike trains can evoke an order-of-magnitude increase in the contractile force produced, despite having average firing frequencies that are subthreshold for a contractile response. This is because SH spike trains exhibit amplified respiratory bursts. We therefore sought to quantify the response of the model to bursting inputs.

### 4.5 Bursting stimulation of model

**Fig. 7:**
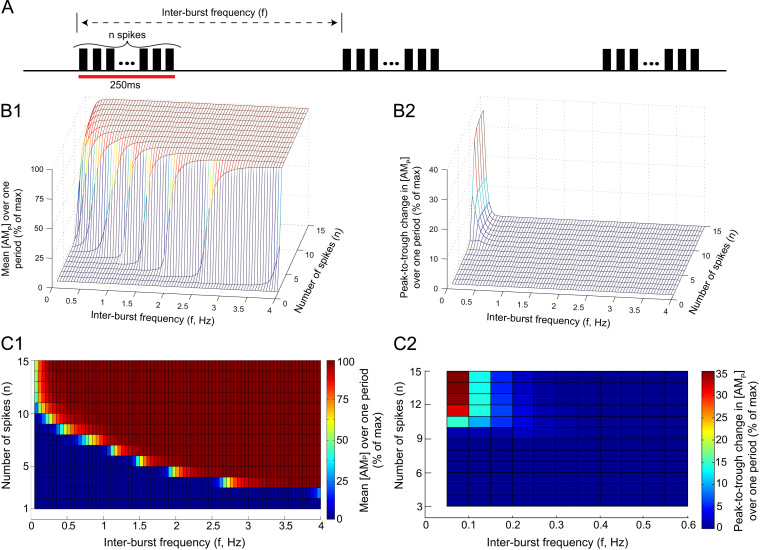
Contractile force critically depends on burst amplitude. (A) The SPGN model was stimulated with bursts of *n* pulses, at a frequency $T^{-1}=f$. (B) For each tuple (*n*, *f*) the response of the contractile force $\left[{\mathrm{AM}}_{P}\right]$ was plotted. (B_*1*_) The mean $\left[{\mathrm{AM}}_{P}\right]$ over one period, as a % of the maximum contractile force. (B_*2*_) The oscillatory response of $\left[{\mathrm{AM}}_{P}\right]$ to the bursting stimulation, measured as the peak-to-trough change in $\left[{\mathrm{AM}}_{P}\right]$ over one period (% of max). An oscillatory response was only seen for very high amplitude (large *n*) bursts, at very low inter-burst frequencies *f*. (C) Heat maps depicting the data for (B). Note that at frequencies mimicking respiratory modulation of sympathetic activity (≈0.4 Hz, as in Fig. 6), $\left[{\mathrm{AM}}_{P}\right]$ is very sensitive to changes in burst amplitude *n*. The magnitude of the [AM_*p*_] response, and any oscillations in [AM_*p*_] produced, is also strongly dependent on *f*, particularly at lower inter-burst frequencies (f<0.4 Hz).

**Fig. 8:**
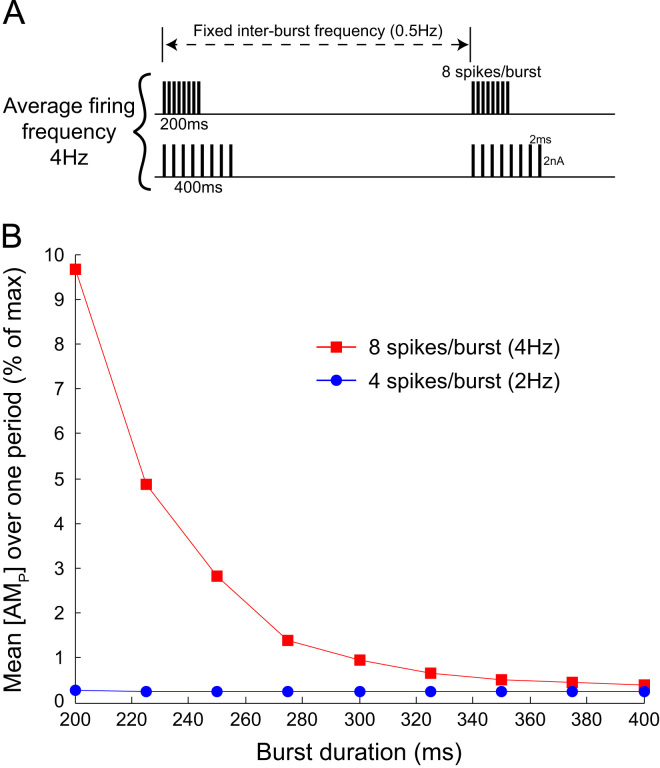
Contractile force depends on burst duration. (A) The SPGN model was stimulated with bursts of fixed amplitude (number of spikes, *n*) and duration. The bursts had a fixed inter-burst frequency of 0.5 Hz, to mimic respiratory modulation of bursting seen *in situ* (Stalbovskiy et al., 2014). Furthermore, burst amplitudes of n=4 and n=8 were chosen to match the burst amplitudes recorded *in situ* in normotensive (control) WKY rats and spontaneously hypertensive rats, respectively (Briant et al., 2014). These patterns achieved average firing rates of 2 Hz and 4 Hz, approximately what was reported in the two strains. These spikes were then equally spread over a burst of duration 200–400 ms. (B) $\left[{\mathrm{AM}}_{P}\right]$ in response to these bursts was recorded at steady-state, after >100s of simulation. For 4 Hz stimulation, the shorter the burst, the greater the contractile response.

To quantify the behaviour of SMCs to bursts, the model was stimulated with bursts of action potentials with varying properties; burst amplitude (spikes/burst, *n*), inter-burst frequency (*f*), and burst duration. These burst parameters were varied over physiological ranges to determine their effect on the contractile force $\left[{\mathrm{AM}}_{P}\right]$. The results are shown in Figs. 7 and 8.

Fig. 7 shows the contractile force $\left[{\mathrm{AM}}_{P}\right]$ generated quantified as a function of the inter-burst frequency *f* and burst amplitude *n*. A series of bursts characterised by fixed *f* and *n* was generated by stimulating the SPGN model with bursts of *n* brief 2 ms pulses in current 2 nA amplitude, incoming at a frequency *f* (Fig. 7A). These pulses were equally spaced across the duration of the burst (250 ms), and induced a burst of *n* action potentials in the SPGN model. The number of spikes *n* was chosen to vary between 1 and 15, covering intra-burst frequencies seen *in situ* for the respiratory components of bursting in normotensive (control) Wistar-Kyoto rats (*n*=2–5) (Stalbovskiy et al., 2014) and spontaneously hypertensive (SH) rats (*n*=6–10) (Briant et al., 2014). In this experimental protocol, the respiratory frequency of the rat is between 0.7 and 1 Hz. The fixed burst duration of 250 ms was chosen to mimic; first, the duration of oscillatory inputs used in studies of sympathetic transmission to the vasculature in conscious rat (Stauss and Kregel, 1996) and; second, the duration of respiratory modulated bursts exhibited by sympathetic preganglionic neurones (SPN) *in situ* (Stalbovskiy et al., 2014; Briant et al., 2014). The inter-burst frequencies *f* ranged between 0.05 and 4 Hz. This pattern of bursting was played in for (>100s), at which steady-state behaviour was reached in the model. The mean contractile force $\left[{\mathrm{AM}}_{P}\right]$ across one period and the amplitude of any oscillations in $\left[{\mathrm{AM}}_{P}\right]$ was recorded, and plot against (*n*, *f*) in a 3D plot (Fig. 7B_1_ and B_2_).

Note how the mean $\left[{\mathrm{AM}}_{P}\right]$ response shows an approximately sigmoidal increase with both increasing frequency *f* and number of spikes/burst *n* (Fig. 7B_1_). As the number of spikes per burst decreases, response against *f* becomes gentler and less sigmoidal. Similarly, as the frequency *f* at which the bursts arrives decreases, function of *n* becomes gentler. Fig. 7C_1_ shows a heat-map for this simulation data. The colour map indicates the level of $\left[{\mathrm{AM}}_{P}\right]$ for each (*n*, *f*). The heat-map shows that the contractile force $\left[{\mathrm{AM}}_{P}\right]$ has a much steeper relationship with *n* than it does with the frequency *f* at which the bursts arrive.

An oscillatory response of $\left[{\mathrm{AM}}_{P}\right]$ to bursting was not seen for all values of *f* and *n* (Fig. 7B_2_). Only bursts incoming at low frequencies (f≤0.3Hz) and with high amplitude (n≥9) were able to induce significant (≥5%) oscillations in $\left[{\mathrm{AM}}_{P}\right]$. When bursts consisting of n>10 spikes where incoming at f=0.05Hz, the SMC was able to produce $\left[{\mathrm{AM}}_{P}\right]$ large oscillations (35% of maximal $\left[{\mathrm{AM}}_{P}\right]$). For inter-burst frequencies f≤0.3Hz oscillations would begin at some critical number of spikes *n*, and rise to a plateau, at which further addition of spikes could not achieve greater oscillatory amplitudes. In Fig. 7C_2_ a heat-map is shown for the oscillatory $\left[{\mathrm{AM}}_{P}\right]$ data. The colour of each (*n*, *f*) square indicates the peak-to-trough change in $\left[{\mathrm{AM}}_{P}\right]$ over one period, according to the colour map.

When bursts are played in at inter-burst frequencies matching respiratory frequencies of 0.5 Hz, as seen in the rat *in situ* (Stalbovskiy et al., 2014; Briant et al., 2014), burst amplitude (*n*) greatly increases the contractile force produced at a critical value of n=8. This critical value is a burst amplitude rarely exhibited by SPN in normotensive control rats, but characteristic of respiratory bursts in SH rats (Stalbovskiy et al., 2014; Briant et al., 2014).

Fig. 8 shows the contractile force $\left[{\mathrm{AM}}_{P}\right]$ as a function of burst duration. For these data, the inter-burst frequency was fixed at *f*=0.5 Hz which is consistent with the frequency of respiratory modulation of SNA *in situ*. Bursts with fixed amplitude *n*=4 and *n*=8 spikes, yielding average firing frequencies of 2 Hz and 4 Hz, respectively, were played into the SPGN model. These burst amplitudes covered average firing frequencies and burst amplitudes seen *in situ* in normotensive (control) WKY rats and SH rats (Stalbovskiy et al., 2014; Briant et al., 2014). As the duration of the burst was decreased, the mean contractile force increased. The contractile force is therefore dependent on the intra-burst frequency.

## 5 Discussion

The aim of this study has been to investigate the sensitivity of arterial smooth muscle cell tone to properties of the burst signals in sympathetic postganglionic neurones. To achieve this we have synthesised and adapted previously published mathematical models (Hai and Murphy, 1988; Destexhe et al., 1994; Li and Rinzel, 1994; Lemon et al., 2003; Bennett et al., 2005; Briant et al., 2014), to produce an *in silico* description of the pathway from action potential generation in a sympathetic postganglionic neurone to the contractile force produced by a SMC. We have for the first time been able to explore in a model the influence of sympathetic patterning on the contractile response of smooth muscle cells.

We found that SMCs are unresponsive to tonic stimulation at average firing frequencies typical of those reported in single sympathetic fibres (Habler et al., 1994), which highlights the importance of bursting. We showed that the SMC was able to generate contractile forces in response to these bursts (compared to tonic stimulation at the same average firing frequency) and that the magnitude of this contractile force is strongly dependent on sympathetic burst properties. In particular, adding 1–2 spikes to a burst could cause the model to generate a contractile response. Given that this increase in burst amplitude is characteristic of sympathetic preganglionic neurones in the spontaneously hypertensive rat, we drove the model with such spike trains. The contractile response generated by the model increased markedly (compared to normotensive control trains) due to potentiated release of NA. These data provides the first mechanistic understanding of how increased respiratory modulation, recorded in the pre-hypertensive SH rat, causes higher vascular resistance and therefore contributes to the pathology of this disease (Simms et al., 2009; Moraes et al., 2014; Briant et al., 2014).

### 5.1 What causes the greater response to bursting?

The impact of irregular or bursting sympathetic stimulation on vasoconstriction has been studied in the rat mesentery (Nilsson et al., 1985), pig nasal mucosa (Lacroix et al., 1988), dog gracilis muscle and pig spleen (Pernow et al., 1989). All studies reported an increased response of the innervated vasculature to irregular or burst patterns (compared to tonic), despite average firing frequencies (all <5Hz) being subthreshold for a mechanical response. Our simulations show that the ability of the vasculature to respond to such low firing rates is achieved because release of NA is potentiated by grouping spikes. Stimulation of our model with spike trains, characterised by grouped stimuli that occur during the inspiratory period (Fig. 6), produced greater contractile forces despite the SMC model being unresponsive to such a low average firing frequency.

Julien et al. (2001) stimulated SMCs with phenylephrine and attributed the frequency-dependent response to NA kinetics, rather than to post-junctional intra-SMC signal processing. The present study supports this, showing that the kinetics of NA release determines the amplified response of the SMC to bursting. In cardiac myocytes, systolic calcium concentration (the amplitude of Ca^2+^ transients) depends cubically on SR content (Trafford et al., 2001). This highly non-linear relationship means that small changes in SR content markedly influence myocyte contractility. Similarly in the present study, the relationship between firing frequency and the amplitude of pre-synaptic calcium transients is highly non-linear. As the frequency increases, the transients achieve greater amplitudes, potentiating NA release (Figs. 4 and 5). This occurs via a non-linearity with activation of fusion protein; the gain in fusion protein activation was seen to be strongly dependent on the intra-burst frequency. The pre-synaptic calcium dynamics are therefore causing the preferential response to bursting; this is supported by experimental data of sympathetic stimulation of small mesenteric arteries (Sjoblom-Widfeldt and Nilsson, 1989). In the study, a moderate reduction in extracellular calcium greatly changed the frequency-response curve, a shift that was due to pre-junctional mechanisms (such as altered NA release from nerve-endings).

### 5.2 Sympathetic bursts are not unitary events

The simulations from the present study show that the properties of a burst are important determinants of the contractile response of SMCs (Figs. 7 and 8). Analysis of muscle sympathetic nerve activity (mSNA) in humans has largely considered bursts in mSNA as unitary events—measuring bursts/min and ignoring the temporal spacing and amplitude of these burst events (Macefield, 2013). This assumption has recently been challenged by the work of Fairfax et al. (2013). They show that individual cardiac-triggered bursts of mSNA can dynamically regulate forearm vascular conductance (FVC), and that both the incoming pattern and magnitude of the bursts are able to decrease FVC by an *α*-adrenoreceptor mediated mechanism. Our data not only corroborates these findings, highlighting the need to move away from considering bursts as unitary events, but quantifies in detail the influence of burst amplitude, pattern and duration on the contractile response of SMCs.

The nature of the contractile response of a single SMC to periodic phenylephrine application is known to be frequency-dependent. Julien et al. (2001) studied SMCs isolated from the aorta of four-week old rats, and demonstrated that they tonically contract when the frequency of application was >0.1Hz, but could produce periodic contractions at lower frequencies. Concordantly, our model shows that only slow (<0.4Hz) bolus application of NA to the SMC model may result in a significant periodic constriction and dilation of a single SMC (Fig. 3B); at higher frequencies of stimulation the model exhibited a maintained contraction. It has been shown that the sympathetic nervous system (SNS) is too ‘sluggish’ to transmit frequencies higher than 1 Hz in rat (see review Julien et al., 2001). Stimulation of the sympathetic nerve with bursts of fixed amplitude (*n*=6) and duration (250 ms), revealed that the peripheral SNS in conscious rats can only transmit oscillations to the vasculature at frequencies <1 Hz—any higher frequencies simply contribute to the mean level of vasoconstriction (Stauss and Kregel, 1996). Contrarily, Fairfax et al. (2013) reported that the absence of a mSNA burst in a cardiac cycle is followed by vasodilation. This data indicates that periodically occurring bursts of mSNA dynamically control vascular conductance on a (heart) beat-by-beat timescale, implying that the vasculature is relatively responsive to changes in mSNA. Our model adheres to these data, as it was only able to produce oscillatory contractions in response to bursting input when f≤0.3Hz (Fig. 7B_1_). However, our simulation data suggests that the ability of the SNS to transmit oscillatory signals to SMCs depends not only on their incoming frequency, but also their magnitude and duration.

### 5.3 Sympathetic bursting and disease

The results of the present study provide insight into the pathology of hypertension. In the rat, the entrainment of the sympathetic outflow to respiration manifests as bursting activity at respiratory frequencies of 0.5 Hz *in situ* (Simms et al., 2009; Stalbovskiy et al., 2014). Simms et al. (2009) have demonstrated that the phrenic-triggered average of SNA is doubled in amplitude and decreased in duration in the spontaneously hypertensive rat. The sympathetic-respiratory coupling is therefore amplified in this strain. Furthermore, in a recent study (Briant et al., 2014), we reported that sympathetic preganglionic neurones in the SH rat display a 40% increase in their average firing frequency (from approximately 2.5 Hz to 3.5 Hz) and an increase of 2–3 spikes per respiratory burst (compared to normotensive controls). These dysfunctions increase respiratory modulation of sympathetic outflow, findings that we have also attributed to increased excitatory respiratory drive to pre-sympathetic neurones in the medulla (Moraes et al., 2014).

In our model, the spike trains from the SH rat were subthreshold for inducing a contractile response, but the respiratory bursts increase NA release and SMC tone. The SH spike trains evoked a 10-fold increase in the contractile response, compared to that evoked by the normotensive train. The amplified respiratory bursts seen in this disease strain would therefore greatly increase SMC tone and contribute to the pathology of high blood pressure.

### 5.4 Model limitations

Our model makes many assumptions about the sympathetic transmission to SMCs, implicit in the theoretical framework. One assumption is that we have played in preganglionic spike trains into a postganglionic model; implicit in this assumption is that there is no pre-to-postganglionic gain. Gain at the level of the sympathetic postganglionic neurones is known to occur due to high-frequency summation of secondary synaptic inputs (Karila and Horn, 2000; Wheeler et al., 2004; Rimmer and Horn, 2010). Given the dependence of secondary gain on high-frequency coincidence and the entrainment of preganglionic activity to respiration, it is reasonable to assume that any gain would occur during the respiratory-modulated burst. Given that the contraction of our SMC model is highly sensitive to changes in burst amplitude (Fig. 7C_1_), any such gain will markedly influence the contractile response of the cell. The function of postganglionic gain, therefore, could be to add spikes at the critical moment—during bursts—in order to generate a meaningful response from the SMCs comprising the artery wall.

Our model only considers the contractile response due to NA release. Co-transmission is known to occur at sympathetic terminals, with the co-release of neuropeptide Y (NPY) and adenosine 5 ${}^{\prime }$-triphosphate (ATP) (Pablo Huidobro-Toro and Veronica Donoso, 2004; Burnstock, 2009). Early during a train of postganglionic action potentials, smooth muscle cell contraction is activated by ATP (Wier et al., 2009). ATP is then eliminated from the receptor area in 50–100 ms (Bao et al., 1993). ATP therefore has a short-lived influence on contraction. Later during a train, contraction is maintained due to the binding of NA to *α*_1_Rs (Wier et al., 2009). Therefore, our model of NA release is likely to provide a good approximation of steady-state contraction of a single SMC to prolonged sympathetic stimulation.

Is the 3.8% increase in SMC tone in response to SH spike trains vs normotensive spike trains biologically significant? This question relates to another aspect of our model—we have modelled the contractile response of a single SMC innervated by a single postganglionic axon. This simplification may explain why the generated contractions from experimental spike trains appear low (<4% of the maximum force available). Considering that SMCs are circumferentially arranged in arteries (Peters et al., 1983; Luff, 1996), a decrease in SMC length translates to a change in arterial radius. As vascular resistance is inversely proportional to the 4th power of this radius (by the Hagen–Poiseuille equation), the small change in SMC length, as seen in response to SH spike trains, can substantially increase vascular resistance. Our future work will model a network of SMCs comprising the arterial wall, innervated by a plexus of sympathetic fibres.

### 5.5 Conclusions

The model has provided a theoretical framework of SMC contraction following stimulation of a sympathetic postganglionic neurone. It provides insight into the dependence of vasoconstriction in arteries on the firing pattern of the neurone. Employment of this model into a macro model of an artery could be used to investigate the influence of altered sympathetic patterning—such as those seen in disease states—on blood pressure. The model predicts that contractility is highly dependent on bursting properties, and that this is due to pre-junctional regulation of NA release. Experimental testing of these predictions is important, as they have implications for the altered sympathetic bursting in disease states, such as hypertension (Simms et al., 2009) and heart failure (Sverrisdottir et al., 2000).

## Figures and Tables

**Table 1:** Model parameter values.

Parameter	Value	Units	References
${\left[{\mathrm{Ca}}^{2+}\right]}_{\mathrm{syn}}$			
*k*_*t*_	10	mMms^−1^	Destexhe et al. (1994)
*K*	5×10^−4^	mM	Destexhe et al. (1994)
*d*	0.1	μm	Destexhe et al. (1994)
*τ*_*r*_	500	ms	Destexhe et al. (1994)
${\left[{\mathrm{Ca}}^{2+}\right]}_{\infty }$	0.01	μM	Destexhe et al. (1994)
*F*_*d*_	9.648×10^4^	Cmol^−1^	Constant

NA release			
*k*_*b*_	1×10^16^	mM^−4^ms^−1^	Yamada and Zucker (1992)
*F*_*max*_	1×10^−3^	mM	Yamada and Zucker (1992)
*k*_*u*_	0.1	ms^−1^	Yamada and Zucker (1992)
*k*_1_	1000	ms^−1^	Yamada and Zucker (1992)
[V]	0.1	mM	Yamada and Zucker (1992)
*k*_2_	0.1	ms^−1^	Yamada and Zucker (1992)
*k*_3_	4	ms^−1^	Yamada and Zucker (1992)
*N*	10,000	1	Destexhe et al. (1994)
*k*_*h*_	0.003	ms^−1^	Stjarne et al. (1994)

*α*_1_R activation			
$\left[R_{T}\right]$	2×10^4^	1	Lemon et al. (2003) and Bennett et al., 2005
*K*_1_	5	μM	Bennett et al. (2005)
*K*_2_	100	μM	Bennett et al. (2005)
*k*_*r*_	1.75×10^−6^	ms^−1^	Lemon et al. (2003) and Bennett et al. (2005)
*k*_*p*_	0.3×10^−3^	ms^−1^	Bennett et al. (2005)
*k*_*e*_	6×10^−6^	*ms*^−1^	Lemon et al. (2003) and Bennett et al. (2005)
*ζ*	0.85	1	Lemon et al. (2003) and Bennett et al. (2005)

**Table 2:** Model parameter values.

Parameter	Value	Units	References
G-protein cascade			
*k*_*deg*_	0.00125	ms^−1^	Fink et al. (1999)
*α*	2.782×10^−12^	ms^−1^	Lemon et al. (2003) and Bennett et al. (2005)
*K*_*C*_	0.4	μM	Lemon et al. (2003) and Bennett et al. (2005)
*r*_*r*_	0.00015	ms^−1^	Lemon et al. (2003) and Bennett et al. (2005)
$\left[{\left({\mathrm{PIP}}_{2}\right)}_{T}\right]$	5×10^7^	1	Lemon et al. (2003) and Bennett et al. (2005)
${\left[{\mathrm{IP}}_{3}\right]}_{0}$	0.02	μM	Lemon et al. (2003) and Bennett et al. (2005)
*N*_*A*_	6.022×10^23^	1	Constant
*k*_*a*_	0.17	s^−1^	Lemon et al. (2003) and Bennett et al., 2005
*k*_*d*_	1.5	s^−1^	Lemon et al. (2003) and Bennett et al. (2005)
*C*_*V*_	5×10^−16^	m^3^	Lemon et al. (2003) and Bennett et al. (2005)

${\left[{\mathrm{Ca}}^{2+}\right]}_{\mathrm{SMC}}$ dynamics			
${\left[{\mathrm{Ca}}^{2+}\right]}_{\mathrm{SR}}$	400	μM	Fink et al. (1999)
*J*_*max*_	2.88	$\mu M\;{\mathrm{ms}}^{-1}$	Fink et al. (1999)
*V*_*max*_	0.00585	$\mu M\;{\mathrm{ms}}^{-1}$	Fink et al. (1999)
*K*_*p*_	0.24	μM	Fink et al. (1999)
*K*_*I*_	0.03	μM	Fink et al. (1999)
*K*_*inh*_	0.1	μM	Fink et al. (1999)
*k*_*on*_	0.008	$\mu M^{-1}\;{\mathrm{ms}}^{-1}$	Fink et al. (1999)
*K*_*act*_	0.17	μM	Fink et al. (1999)
*P*_*L*_	0.394×10^−3^	$\mu M\;{\mathrm{ms}}^{-1}$	Fink et al. (1999)
*β*	1/41	1	Fink et al. (1999) and Bennett et al., 2005
*δ*	0.0043	μM	Fink et al. (1999)

Cross-bridge			
$\left[M_{T}\right]$	2	μM	Yagi et al. (1988)
k^m	8×10^−3^	$\mu M^{-4}\;{\mathrm{ms}}^{-1}$	Bennett et al. (2005)
k^2	0.5×10^−3^	ms^−1^	Hai and Murphy (1988)
k^3	0.4×10^−3^	ms^−1^	Hai and Murphy (1988)
k^4	0.1×10^−3^	ms^−1^	Hai and Murphy (1988)
